# Dropout among patients in qualified alcohol detoxification treatment: the effect of treatment motivation is moderated by Trauma Load

**DOI:** 10.1186/1747-597X-8-14

**Published:** 2013-03-21

**Authors:** Michael Odenwald, Peter Semrau

**Affiliations:** 1 Department of Psychology, University of Konstanz, Universitätsstr. 10, Konstanz 78464, Germany; 2 Forel Clinic, Islikonerstrasse 58548 Ellikon an der Thur, Switzerland, Previously: Center for Psychiatry Reichenau, Feursteinstr. 55, Reichenau 78479, Germany

## Abstract

**Background:**

Motivation to change has been proposed as a prerequisite for behavioral change, although empirical results are contradictory. Traumatic experiences are frequently found amongst patients in alcohol treatment, but this has not been systematically studied in terms of effects on treatment outcomes. This study aimed to clarify whether individual Trauma Load explains some of the inconsistencies between motivation to change and behavioral change.

**Methods:**

Over the course of two months in 2009, 55 patients admitted to an alcohol detoxification unit of a psychiatric hospital were enrolled in this study. At treatment entry, we assessed lifetime Trauma Load and motivation to change. Mode of discharge was taken from patient files following therapy. We tested whether Trauma Load moderates the effect of motivation to change on dropout from alcohol detoxification using multivariate methods.

**Results:**

55.4% dropped out of detoxification treatment, while 44.6% completed the treatment. Age, gender and days in treatment did not differ between completers and dropouts. Patients who dropped out reported more traumatic event types on average than completers. Treatment completers had higher scores in the URICA subscale Maintenance. Multivariate methods confirmed the moderator effect of Trauma Load: among participants with high Trauma Load, treatment completion was related to higher Maintenance scores at treatment entry; this was not true among patients with low Trauma Load.

**Conclusions:**

We found evidence that the effect of motivation to change on detoxification treatment completion is moderated by Trauma Load: among patients with low Trauma Load, motivation to change is not relevant for treatment completion; among highly burdened patients, however, who a priori have a greater risk of dropping out, a high motivation to change might make the difference. This finding justifies targeted and specific interventions for highly burdened alcohol patients to increase their motivation to change.

## Background

Dropout rates from detoxification treatment are high and the reasons for this are poorly understood [1-4]. Based on Prochaska’s & Di Clemente’s original work on the Transtheoretical Model (TTM) of Behavior Change [5], changes in addictive behaviors occur in five stages with specific motivational characteristics: starting to thinking about change (Precontemplation), weighting reasons to change or not to change (Contemplation), planning to change (Preparation), trying and realizing first changes (Action) and maintaining achieved changes (Maintenance) [6]. The model is dynamic and assumes that behavioral change is a process, with individuals repeatedly cycling through these stages and between abstinence and relapse. The process thus involves learning and personal growth before a stable abstinence is achieved. Based on the current specific stages an individual’s readiness to change can be derived at any point in time [6]. This model has inspired addiction science since its inception, although studies that link motivation to change to treatment outcome are inconsistent: in some studies, stages of the TTM and readiness to change upon starting treatment predict treatment outcome [7-9], while other researchers argue that motivation to change is not related to behavioral change [10,11].

Numerous studies have shown manifold ways of how Posttraumatic Stress Disorder (PTSD) or traumatic experiences are associated with the onset, course, maintenance and therapy of alcohol problems: for example, there is a large co-morbidity of lifetime alcohol use disorders among PTSD patients (e.g. ex-combatants [75%; [12] and the general population [52%; [13]). Additionally, a large proportion of patients in alcohol treatment units has experienced traumatic events or suffers from PTSD (PTSD rate: 15-34%; [14,15]). Substance-dependent patients with PTSD generally have a worse physical and psychological health status as well as higher disability ratings than those without PTSD [16,17]. Furthermore, fluctuations of PTSD and substance dependence symptoms in untreated subjects are highly correlated [17]. Growing evidence supports an etiological link between PTSD and the subsequent development of substance use disorders in terms of a functional use to normalize or “selfmedicate” trauma-related psychiatric symptoms [18,19]. Neuro-pharmacological studies support the notion that alcohol is effective for suppressing PTSD-related hyperarousal and emotional symptoms and that alcohol withdrawal can reactivate PTSD symptoms, for example, through CRH excretion [20]. Regarding the treatment of alcohol disorders, recent studies have identified PTSD as a risk factor for substance use relapse and a poorer treatment outcome [21] whereas patients with double diagnosis who received PTSD treatment had a better addiction-related long-term outcome than those who did not receive treatment [22]. These studies show that traumatic experiences and PTSD are important variables that need to be taken into consideration when studying the motivation underlying behavioral change in alcohol treatment. Although no study directly targeted this issue, certain recent studies indeed lend first support to this notion as they showed that readiness to change alcohol use at treatment entry is negatively correlated with emotional distress [23] and that non-addictive psychopathology is a predictor of motivational changes [6]. Based on our previous studies with traumatized alcohol patients [24], we believe that patients with high levels of posttraumatic stress symptoms find detoxification treatments extremely aversive since the reality of acute wards frequently provokes intrusive memories and hyperarousal; they therefore drop out in order to avoid traumatic symptom recurrence. In our experience, those alcohol patients with PTSD who have a high motivation to change will be better able to tolerate and complete detoxification. However, a systematic research into how trauma experiences are related to one’s motivation to change is missing.

We studied whether Trauma Load and motivation to change interact with treatment outcome; i.e. treatment completion or treatment dropout. We used Trauma Load as a proxy for posttraumatic stress symptoms, as PTSD could not be measured in this study. We theoretically see Trauma Load as a moderator variable; according to Baron and Kenny [25] “moderation implies that the causal relation between two variables changes as a function of the moderator variable” (p.1174). In this study, we tested three hypotheses: 1) patients who completed treatment had a higher motivation to change at treatment entry and 2) (to replicate existing findings) that dropout is related to a higher Trauma Load. The main hypothesis (3) is that Trauma Load interacts with the motivation to predict treatment completion - that is, that the association between one’s motivation to change and treatment completion is different between patients with high and low Trauma Load.

## Methods

### Design

This prospective study was implemented in an alcohol detoxification unit of a public psychiatric hospital in southern Germany with 29 in-patient beds. The unit has approximately 800 admissions per year. In 2009, the average treatment length was 13.4 days (SD 10.2). This study was conducted as part of a larger research project, which also included an intervention study [24] that, however, did not include the analysis of treatment motivation. During the two month-long recruitment period for this study, all newly admitted patients were asked to participate in the study. We excluded all patients who were assigned to the intervention group of the aforementioned treatment study that was done so in order to enhance treatment retention [24]. However, patients who participated in the control group (Treatment as Usual) were admitted; in total, 58% of the sample studied here participated in the control arm and 42% were not involved in the other study (e.g. they were not eligible for the other study).

On the day of admission or soon afterwards (when intoxication or severe withdrawal symptoms had ended), nursing staff members informed the patient of the study. Patients received written information and a consent form to declare their voluntary study participation. If patients agreed to participate, nursing staff delivered the questionnaires and asked the patients to complete them. Completed questionnaires were returned to the nursing staff. Participants received no monetary compensation.

### Measurements

The questionnaires were comprised of socio-demographic questions, the German version of the Trauma History Questionnaire (THQ) and the German short form of the University of Rhode Island Change Assessment (URICA).

The THQ [26,27] is a frequently used list of 24 stressful and traumatic event types, such as being exposed to violent assault, an accident or rape. The respondent indicates whether he or she has experienced each event type during their lifetime and if so, at which age they first experienced it. A test-retest study confirmed the stability of the self-reported THQ items [26]. Based on the information regarding how many of the THQ event types had ever been experienced, we computed a sum score (range 0 to 24). This 24-item THQ event list achieved a satisfactory internal consistency (Cronbach’s alpha .80). In this study, we used the THQ sum score as a moderator variable for treatment motivation as posttraumatic stress disorder and other disorders cannot be reliably assessed during alcohol withdrawal [28]. The number of experienced traumatic events has been shown to be one of the best predictors of PTSD and other post-traumatic psychopathology in the sense of a dose-effect relationship [29].

Motivation to change was assessed using the German short form of the University of Rhode Island Change Assessment (URICA) [30,31], the “Veränderderungsstadienskala“ (VSS-K) [32]. Using 16 of the original 32 items [30] that had been translated into German, the VSS-K assesses four motivational components based on Prochaska’s and DiClemente’s model, each with four items: Precontemplation (e.g., “I guess I have faults but there’s nothing that I really need to change”), Contemplation (e.g., “I have a problem and I really think I should work on it”), Action (e.g., “I am actively working on my problem”) and Maintenance (e.g., “I may need a boost right now to help me maintain the changes I’ve already made”). The answer format is a five-point Likert scale, with higher values indicating more endorsement of the respective behavior. For the VSS-K, weak to moderate coefficients of internal consistency (Cronbachs Alpha: Precontemplation .63, Contemplation .56, Action .78, Maintenance .79) and satisfactory validity, such as the replication of the factorial structure of the English instrument, are reported [33]. The VSS and its brief version, the VSS-K, are frequently used measures of the motivation to change within the German language area; the authors of the German version recommend using the four subscales of the instrument to quantify dimensions of motivation and not as categorical stages. In addition to the four VSS-K/URICA subscales, we used the composite Readiness To Change (RTC) score, which is calculated by adding the three subscale scores of Contemplation, Action and Maintenance and subtracting the Precontemplation subscale score [34,35].

We also assessed circumstances of discharge and length of treatment, as recorded in the patients’ hospital files. Circumstances of discharge were coded as “dropout” when patients ended treatment “against medical advice” or when the clinic ended the treatment because of the patient (repeatedly) breaching rules (“disciplinary”). “Treatment completion” was coded when patients completed treatment (“regular”) and when they were “transferred to subsequent treatment” (i.e. psychotherapy units within or outside the clinic).

### Participants

In total, 159 patients were admitted to the ward during the research project. Of them, 37 were excluded because they participated in a group intervention to improve treatment adherence. Of the remaining 122, 22 did not provide their consent. We have no data for this group. Among the 100 who provided their consent to participate in the study, no information on treatment completion or dropout are available for five, while 40 did not return the questionnaires (39 returned neither questionnaire, 1 returned only the THQ). For the latter, we can, however, report socio-demographic data. 55 participants had retuned both questionnaires, although some left parts of their questionnaires blank, so that N still varies in different analyses. Based on 122 potential participants, the de facto participation rate was 45,1%. Comparing the 55 participants who returned the THQ and the VSS-K/URICA with the 40 who did not, we did not find differences in respect to treatment dropout (Chi^2^ = 1.046, df = 1, p = .306), gender (Chi^2^ = .279, df = 1, p = .597), age (T = −.712, df = 92, p = .479) and duration of treatment (T = .266, df = 93, p = .869).

Socio-demographic characteristics of included participants are shown in Table 1.

**Table 1:** Characteristics of participants

	**Total (55)**	**Completers (25)**	**Dropouts (30)**	**Test statistic (p)**
Age^1^	43.65 (8.77)	41.58 (9.76)	45.30 (7.66)	1.569^2^ (.123)
Gender female	26.8% (15)	32.0% (8)	23.3% (7)	.516^3^ (.472)
Days in treatment	13.98 (10.68)	13.64 (10.85)	14.27 (11.24)	-.254^4^ (.799)

^1^ Missing data: N = 54, 30 dropouts, 24 completers.

^2^ t-test, df 52.

^3^ Chi^2^-test, df 1.

^4^ Wilcoxon’s test, Z-score reported.

We report mean (SD) and percentages (N).

### Ethical approval

The University of Konstanz Research Review and Ethics Board approved the study.

### Data analysis

Data were analyzed using SPSS version 20 for Mac. We report means, standard deviations and percentages. We relied on Fisher’s exact test when preconditions of Chi^2^ tests were not fulfilled when comparing percentages. Group differences of quantitative variables were tested using ANOVAs and Student’s t-tests; if preconditions were not fulfilled, we used Wilcoxon’s tests or Kruskal-Wallis tests. Preconditions for parametric statistical methods were tested using the Levene’s test (homoscedasticity) and the Kolmogorov-Smirnov test (normal distributions). All variables were normally distributed except for days in treatment. Homoscedasticity was not violated. We report two-tailed p values. Alpha was set to 0.05.

We used the median split to divide patients into groups with high (28) and low Trauma Load (27) as well as into groups of high and low VSS-K/URICA subscale scores (Ns reported in results’ section). We tested the interaction Trauma Load * VSS-K/URICA subscales by cross-tabulating median split independent variables (THQ sum, VSS-K/URICA subscales) and treatment completion vs. non-completion in four 2 * 2 * 2 tables. Furthermore, assuming Trauma Load would be a moderator between motivation to change and treatment completion, we expected different magnitudes of correlations between these variables when comparing patients with high and low Trauma Load. For the comparison of correlations from two independent subgroups we used Fisher’s r-to-z transformation and computed Fisher’s Z-statistic [36].

We report correlations between the variables of interest – namely, Pearson correlations between continuous variables and point-biserial correlations between binary and continuous variables. The assumption that Trauma Load acts as a moderator can be tested in the framework of Baron and Kenny’s moderator approach [25]. The authors state that it is desirable that the moderator variable be uncorrelated with both independent and dependent variables. We found low correlations (r ≤ .151; p ≥ .270) between Trauma Load and the VSS-K/URICA subscales and the RTC composite score as well as a small to moderate correlation with treatment completion (r = .320, p = .017).

We separately analyzed the interaction of Trauma Load with single VSS-K/URICA subscales with two-way ANOVAs and subsequent post-hoc testing.

Because time in treatment showed a complex relationship to Trauma Load, the precondition of the Cox proportional hazard regression of time-independent effects was violated; the inclusion of interaction terms into Cox models additionally leads to difficulties in interpreting results. Thus, we did not rely on survival analysis in our multivariate analysis. Instead, we used binary logistic regression to study the effects of motivation to change (independent variable) and Trauma Load (moderator variable) on completion of detoxification treatment (dependent variable), controlling for socio-demographic variables. We successively forced blocks of theory-founded predictor variables into four nested models. We entered age and gender into model 1, the VSS-K/URICA subscale scores into model 2 and the sum score of the THQ into model 3. In model 4, we added all two-way interaction terms between the moderator and the VSS-K/URICA scales. We used likelihood-ratio tests to assess the goodness of fit (GoF) of each model against the model with constant only. In order to better interpret the results, we centered all continuous predictor variables at their respective mean; in order to include all subjects, we replaced one missing value in the variable age with the sample mean in this analysis. The final model was constructed using a backward inclusion model of all predictor variables in all blocks based on the Wald statistic (inclusion p < .05, exclusion p < .05). In order to be able to interpret interaction terms, we added lower-level variables into the final model in order to achieve a hierarchically well formulated model [37]. In order to facilitate the interpretation of results, we chose not to include the RTC score into the binary regression model, as this represents aggregated information of the four subscales.

## Results

Thirty patients (54.5%) dropped out of detoxification treatment and 25 (45.4%) were completers. Both groups did not differ in respect to age and gender (see Table 1). Completers and dropouts also did not differ in relation to days in treatment (see Table 1).

### Trauma experiences

On average, respondents reported 7.2 (SD 4.38) THQ event types. The most frequently reported events were learning about the unexpected death of a close person, seeing dead bodies in situations other than funerals, being in a life-threatening situation, witnessing death or injury, having something stolen, experiencing a severe accident and experiencing an armed assault (see Table 2).

**Table 2:** Frequency of experienced event types of the Trauma History Questionnaire

	**Total (55)**	**Completers (25)**	**Dropouts (30)**	**Test statistic (p)**
Armed robbery (THQ1)	34.5% (19)	20.0% (5)	46.7% (14)	4.288^1^ (.038)
Something stolen (THQ2)	49.1% (27)	28.0% (7)	66.7% (20)	8.158^1^ (.004)
Burglary when not at home (THQ3)	25.5% (14)	16.0% (4)	33.3% (10)	2.159^1^ (.142)
Burglary when at home (THQ4)	5.5% (3)	0% (0)	10.0% (3)	Fisher’s exact (.242)
Severe accident (THQ5)	47.3% (26)	40.0% (10)	53.3% (16)	.973^1^ (.324)
Natural disaster (THQ6)	12.7% (7)	12.0% (3)	13.3% (4)	Fisher’s exact (1.000)
Man-made disaster (THQ7)	18.2% (10)	12.0% (3)	23.3% (7)	Fisher’s exact (.318)
Environmental poisons/Radiation (THQ8)	23.6% (13)	20.0 (5)	26.7% (8)	.336^1^ (.562)
Other situation with severe damage (THQ9)	27.3% (15)	24.0% (6)	30.0% (9)	.247^1^ (.619)
Life-threatening situation (THQ10)	52.7% (29)	40.0% (10)	63.3% (18)	2.979^1^ (.084)
Witnessed death or injury (THQ11)	50.9% (28)	40.0% (10)	60.0% (19)	2.183^1^ (.140)
Seeing dead bodies (THQ12)	54.5% (30)	32.0 (8)	73.3% (22)	9.396^1^ (.002)
Friend or family member killed (THQ13)	7.3% (4)	8.0% (2)	6.7% (2)	Fisher’s exact (1.000)
Spouse or child died (THQ14)	18.2% (10)	20.0% (5)	16.7% (5)	Fisher’s exact (1.000)
Life-threatening illness (THQ15)	34.5% (19)	40.0% (10)	30.0% (9)	.603^1^ (.437)
Learned about unexpected death (THQ16)	78.2% (43)	72.0% (18)	83.3% (25)	1.027^1^ (.311)
Combat exposure (THQ17)	7.3% (4)	4.0% (1)	10.0% (3)	Fisher’s exact (.617)
Forced sex (THQ18)	12.7% (7)	16.0% (4)	10.0% (3)	Fisher’s exact (.689)
Private body parts being touched (THQ19)	30.9% (17)	32.0% (8)	30.0% (9)	.026^1^ (.873)
Other situation unwanted sex (THQ20)	10.9% (6)	8.0% (2)	13.3% (4)	Fisher’s exact (.678)
Armed assault (THQ21)	38.2% (21)	20.0% (5)	53.3 % (16)	6.419^1^ (.011)
Severe physical assault without weapon (THQ22)	21.8% (12)	16.0% (4)	26.7% (8)	.910^1^ (.340)
Severe beatings by family member (THQ23)	34.5% (19)	24.0% (6)	43.3% (13)	2.254^1^ (.133)
Other event (THQ24)	23.6% (13)	24.0% (6)	23.3% (7)	.003^1^ (.954)

^1^ Chi^2^-test, df = 1.

We report percentages and Ns (in brackets).

Patients who dropped out reported an average more traumatic event types compared to completers (Table 3). Dropouts were more likely to experience armed robbery, armed assault, the stealing of property and seeing dead bodies (Table 2).

**Table 3:** Outcome variables and comparison between completers and dropouts

	**Total (55)**	**Completers (25)**	**Dropouts (30)**	**Test statistic (p)**
THQ-Sum	7.20 (4.38)	5.68 (3.84)	8.47 (4.46)	2.455^2^ (.017)
Age upon experiencing first trauma^1^	15.23 (9.28)	16.09 (9.14)	14.57 (9.48)	-.588^3^ (.559)
Precontemplation	6.01 (2.16)	5.80 (2.24)	6.20 (2.11)	.682^2^ (.498)
Contemplation	15.49 (3.05)	16.12 (2.99)	14.97 (3.06)	−1.407^2^ (.165)
Action	14.85 (3.20)	14.76 (3.59)	14.93 (2.90)	.198^2^ (.844)
Maintenance	13.22 (3.52)	14.24 (3.14)	12.37 (3.64)	−2.020^2^ (.048)
Readiness to Change	37.55 (8.67)	39.32 (8.36)	36.07 (8.79)	−1.397^2^ (.168)

^1^ Missing data: N = 54, 30 dropouts, 23 completers.

^2^ t-test, df 53.

^3^ t-test, df 52.

We report mean (SD) and p values.

Among patients with high Trauma Load, treatment dropout occurred in 64.3% (18/28) and in 44.4% of those with low Trauma Load (12/27; Chi^2^ = 2.183, df = 1, p = .140).

### Comparing treatment motivation between completers and dropouts

In Table 3, the means and SD of the four VSS-K/URICA subscales and of the composite Readiness to Change score are reported. When testing the difference between completers and dropouts, only the Maintenance subscale revealed a small but significant difference – that is to say, completers had a higher average score.

### Interaction Trauma Load * Motivation to predict treatment completion

**Figure 1:**
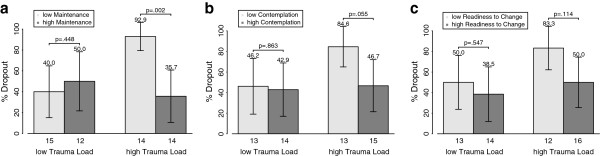
**Dropout rates in groups of patients.** We report treatment dropout in percent and 95% confidence intervals in the four groups with high and low Trauma Load divided by high and low motivation score. Numbers below columns represent numbers of subjects in the respective groups. **A**) Maintenance subscale. **B**) Contemplation subscale, **C**) RTC composite score.

We first tested this interaction by comparing the rate of dropout in the four subgroups of patients with high vs. low VSS-K/URICA subscale and RTC scores by high vs. low Trauma Load (2 * 2 * 2 table; Figure 1). Only the Maintenance and Contemplation subscales revealed a pattern clearly distinct from the random distribution of dropouts. The dropout rate among patients with low Trauma Load was 40% for those with a low and 50% among those with a high Maintenance score (Chi^2^ = .270, df = 1, p = .603). However, among patients with high Trauma Load almost all with a low Maintenance score dropped out (13/14, i.e. 92.9%), compared to just 35.7% of those with a high Maintenance score (Chi^2^ = 9.956, df = 1, p = .002). The Chi^2^ test for the overall effect was not significant (Chi^2^ = 2.979, df = 1, p = .084). A high but non-significant percentage of dropouts was also found amongst the group of high Trauma Load and low Contemplation score (following the order of appearance as above 46.2%, 42.9%, Chi^2^ = .030, df = 1, p = .863; 84.6%, 46.7%, Fisher’s exact test, p = .055; overall effect Chi^2^ = 2.337, df = 1, p = .126). A high but equally non-significant elevation of dropouts was found among patients with high Trauma Load and low Readiness to Chance score (i.e., 50.0%, 38.5%, Chi^2^ = .363, df = 1, p = .547; 83.3%, 50,0% Fisher’s exact test, p = .114; overall effect Chi^2^ = 2.337, df = 1, p = .126). The overall effects of the other subscale models were non-significant (Precontemplation Chi^2^ = .875, df = 1, p = .349; Action Chi^2^ = 0.22, df = 1, p = .883).

Correlations of predictor variables are reported in Table 4. Magnitudes were low, except for the correlations between the three subscales of the VSS-K/URICA. Based on the assumption that the association between predictor and dependent variable is different for different levels of the moderator variable, we separately analyzed the correlations between the VSS-K/URICA scales and treatment completion for patients with high and low Trauma Load (see Table 5 for results). All correlations were moderate to low. The respective differences between the two related coefficients were significant for the Maintenance subscale as well as for the composite Readiness to Chance score. We also found that the direction of associations varied systematically.

**Table 4:** Correlations between predictor variables

	**Age**	**Gender**	**THQ**	**Precontemplation**	**Contemplation**	**Action**	**Maintenance**
Age^1^	-	-.135 (.332)	-.143 (.303)	.131 (.344)	-.014 (.922)	.100 (.473)	-.051 (.716)
Gender		-	-.085 (.539)	-.186 (.174)	.059 (.669)	.075 (.587)	-.015 (.914)
THQ			-	-.004 (.975)	-.017 (.901)	.151 (.270)	-.038 (.785)
Precontem plation				-	-.193 (.159)	-.080 (.561)	-.249 (.066)
Contem plation					-	.458 (< .001)	.720 (< .001)
Action						-	.299 (.027)

1 missing data; N = 54.

We report correlations and p-values (in brackets). N = 55 if not otherwise stated.

**Table 5:** Correlational associations between VSS-K/URICA subscales and treatment outcome in all patients and in subgroups with high and low Trauma Load

	**Total (55)**	**Low Trauma load (27)**	**High Trauma Load (28)**	**Test statistic (p)**
Precontemplation	-.093 (.498)	.073 (.717)	-.320 (.097)	1.416^1^ (.157)
Contemplation	.190 (.165)	-.092 (.648)	.380 (.046)	−1.723^1^ (.085)
Action	-.027 (.844)	-.159 (.429)	.130 (.509)	−1.019^1^ (.308)
Maintenance	.267 (.048)	-.101 (.615)	.515 (.005)	−2.348^1^ (.019)
Readiness to Change	.189 (.168)	-.160 (.425)	.453 (.015)	−2.274^1^ (.023)

^1^ Fisher’s Z.

We report point-biserial correlations and p (in brackets) as well as outcomes of tests of differences between the correlations in the two subgroups.

**Figure 2:**
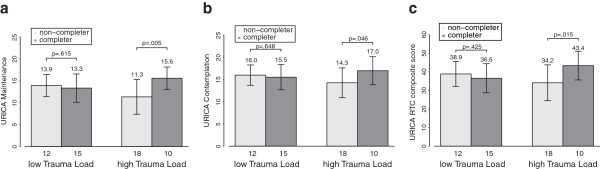
**Interaction effect of Trauma Load and VSS-K/URICA scores on treatment completion.** We report mean and standard deviations of continuous VSS-K/URICA scores. Numbers below columns represent numbers of subjects in the respective groups. **A**) Maintenance subscale. **B**) Contemplation subscale, **C**) RCT composite score.

In order to further illustrate this effect, we plotted mean and SD of the VSS-K/URICA subscales against Trauma Load and treatment outcome (see Figure 2). Using two-way ANOVAs with single VSS-K/URICA scores as the dependent variables we obtained a significant main effect of treatment completion in the Maintenance model (F = 4.173, df1 = 1, df2 = 51, p = .046), a non-significant main effect of Trauma Load (F = .0.31, df1 = 1, df2 = 51, p = .861) and a significant interaction effect (treatment completion * Trauma Load; F = 7.235, df1 = 1, df2 = 51, p = .010; corrected R^2^ = .142). In post-hoc tests, Maintenance was greater among completers than dropouts in the high Trauma Load group (Wilcoxon: W = 204,00, z = −2.767, p = .005) but not in the low Trauma Load group (T = .510, df = 25, p = .615). A significant interaction effect in the same direction was also found for the composite Readiness to Chance score (F = 6.403, df1 = 1, df2 = 51, p = .015, corrected R^2^ = .093; post-hoc: high Trauma Load T = −2.594, df = 26, p = .015; low Trauma Load T = .811, df = 25, p = .425). A nonsignificant interaction effect that was approaching significance was found for the Contemplation subscale (F = 3.761, df1 = 1, df2 = 51, p = .058; corrected R^2^ = .051; post hoc: high Trauma Load T = −2.097, df = 26, p = .046; low Trauma Load T = .462, df = 25, p = .648). No interaction effects could be found with the other subscales of the VSS-K/URICA as dependent variables (Precontemplation F = 1,954, df1 = 1, df2 = 51, p = .168, corrected R^2^ = −.001; Action F = 1.063, df1 = 1, df2 = 51, p = .307, corrected R^2^ = −.023). No main effects were found except for those reported in the Maintenance model reported above.

The binary logistic regression models are reported in Table A1 (Additional file 1). The more complex the model, the higher the variance component that could be explained (up to > 50%). The only significant predictors in the backward inclusion model were Trauma Load (OR .800, CI 95% .670 - .956, p = .014) and the interaction term Trauma Load*Maintenance (OR 1.050, CI 95% 1.003 . 1.098, p = .036). In order to be able to interpret the interaction term, we added Maintenance as lower-order variable [37], which was not significant (OR 1.184, CI 95% .972 – 1.443, p = .094). With this inclusion, the OR of the other variables are slightly changed (Trauma Load: OR .783, CI 95% .645 - .951, p = .014; Trauma Load*Maintenance: OR 1.047, CI 95% .997 – 1.100, p = .065; constant: OR .665, p = .241). The final model accounted for 30% of the dependent variable’s variance. This result means that an increase of one traumatic event type leads to a reduction in the predicted odds to complete treatment by a factor of .78 when Maintenance is kept at its mean. The OR of the interaction term means that at different levels of Trauma Load, the prediction of treatment completion by Maintenance changes; when Trauma Load increases one unit, the OR of Maintenance to predict treatment completion increases by the factor of 1.047, i.e. the higher the Trauma Load levels the stronger the association between Maintenance and treatment outcome.

## Discussion

In this prospective study, patients undergoing alcohol detoxification reported their motivation to change and lifetime Trauma Load at treatment entry and we studied whether these variables predicted the completion of treatment. Simple group comparisons showed that only the Maintenance component of motivation to change at treatment entry was higher in treatment completers and that patients who dropped out of treatment had a significantly higher Trauma Load. While these simple comparisons in the total sample represent moderate effect sizes, we found a clear interaction between Trauma Load and motivation to change: dropout was only predicted by the Maintenance subscale of the VSS-K/URICA - and to a lesser extent by the RTC composite scale- among those patients with high Trauma Load. That is, the higher the motivation, the better the chance for subsequent therapy completion. As predicted, this association was not found among patients with low Trauma Load. In the final logistic regression model, the odds ratio 1.047 of the interaction effect means that a one-unit increase of the Maintenance subscale leads to a significantly different increase in the odds for treatment completion when comparing it at different levels of Trauma Load. When depicting the result (Figure 2) it can be noted that higher levels of Trauma Load lead to a growing increase of the association between predictor (Maintenance) and dependent variable (treatment completion) - by the factor 1.047. Given the fact that Trauma Load is a continuous variable (mean 7.2, range from 0 to 19), the multiplying factor can theoretically determine an increase of this association of up to 139% comparing those with the lowest and highest Trauma Load. The reported results support the hypothesis that Trauma Load moderates the relationship between motivation to change and treatment completion, therefore, the potential causal relation between motivation and behavior in addiction treatment changes as a function of Trauma Load.

Other studies relating motivation to change with treatment outcomes have reported inconsistent results. This study emphasizes one important point: our results support that, in addiction therapy, personal characteristics determine the degree to which motivation predicts behavior. The moderator approach opens up new possibilities for studying the effect of treatment motivation and other theoretically relevant predictors of treatment success: patient characteristics matter in the attempts to improve treatment for substance-use disorders.

Our findings revealed that especially the motivational component Maintenance (and, to a smaller extent, Contemplation) is related to treatment completion. Maintenance, as measured by the VSS-K/URICA, encompasses the motivation to avoid drawbacks and to secure the behavioral changes that have already been achieved. Moreover, it also includes a positive attitude towards treatment (in this case detoxification) – seeing this as chance and a means of assistance to reach or maintain one’s own goals. In the lives of alcohol-dependent patients, long-term abstinence is often only achieved after repeated attempts to quit followed by relapses. Alcohol patients with a high score on the Maintenance scale seem advanced in this learning development because they appear to have realized that they cannot manage this on their own and that they need to accept therapeutic and medical assistance. The Contemplation component of the VSS-K/URICA contains a general acceptance of alcohol dependence as a problem as well as a positive view of therapeutic and medical assistance. In contrast, the Action component, which is not related to positive outcomes in this study, does not explicitly contain this positive attitude towards professional assistance, but rather the focus on one’s own attempts to solve the problem. This difference might explain why the statistical association of treatment completion with Action - in theory, a positive motivational component - diverges from its association with Maintenance and Contemplation. This also explains why the composite RTC score is not better for predicting outcome than Maintenance and Contemplation. The multivariate model construction reported above furthermore shows that single motivational components of the TTM (e.g. Maintenance) cannot be seen in isolation of the other motivational components, since, as seen for example in Model 4 (Additional file 1), the association of Action and the dependent variable was also revealed to be modified by the moderator. Based on this, we can assume that the different motivational components are also not independent from each other in their relationship to the moderator and thus need to be studied together.

The main finding of our study is that Trauma Load moderates the aforementioned motivational components of Maintenance and Contemplation – that is, only in the subgroup of patients with high Trauma Load are these components associated with detoxification treatment completion. In order to understand this moderation effect, we need to have a closer look at the subgroup with high Trauma Load and find out how they differ from the other patients. It is well documented that high Trauma Load among alcohol patients is related to a higher prevalence of comorbid disorders [38]. From psychiatric research it is known that individuals who greatly suffer from psychiatric disorders are more likely to seek treatment [39]. Our previous finding with this group supports this: alcohol patients with high Trauma Load have significantly shorter periods of time between the onset of regular drinking and their first alcohol treatment - approximately five years [40]. We thus believe that high Trauma Load among alcohol patients might be related to higher suffering as well as a higher subjective need for and acceptance of treatment. The Transtheoretical Model and the VSS-K/URICA measure motivational tendencies towards behavioral change. Therefore, the motivational components measured in this study might not just be related to addiction but rather to all problems, including addiction. This might be one aspect that explains why the patients with a higher Trauma Load have a higher motivation to change. However, high Trauma Load is a two-edged factor because it might at the same time increase the risk of dropout: we previously observed that inpatient alcohol detoxification is more stressful to those who are burdened by high Trauma Load, as, for instance, the crowded and sometimes chaotic detoxification wards might frequently trigger trauma-related intrusions. In turn, these individuals are more likely to drop out [24]. Especially among these patients at risk, a low motivation to change (i.e. less awareness that the current detoxification is a form of assistance to achieve one’s goals), might be related to lesser ability to tolerate this kind of stress. Other studies have also found that comorbid psychopathology constitutes a risk for premature termination of alcohol detoxification [41] and less favorable treatment outcomes [42]. However, our results might also indicate that the dropout risk of a high Trauma Load might be compensated by a high motivation to change, for example, when a patient has already started to change (e.g. by reducing alcohol use or integrating traumatic experiences) and wants to maintain this initial success (Maintenance) he or she might better tolerate the stress of detoxification. Another study has additionally found that only those patients with a high motivation to change at treatment entry profit from motivational interventions [43].

Based on our findings several clinical recommendations for alcohol detoxification can be proposed. Our results suggest that motivation enhancement strategies might be useful tools for improving detoxification treatment completion, as shown by certain recent studies [44]. However, they also question the current thinking that all patients need the same form of assistance. Other researchers have also concluded that detoxification treatment needs to pay more attention to vulnerable groups by applying individualized interventions [2,41]. Several studies have recently reported that specific psycho-educational group interventions on stress, trauma and alcohol use increase addiction treatment completion, especially among traumatized patients [24,45]. This approach needs further empirical support. Expanding upon this approach, patients who are at high risk of dropping out of detoxification might additionally profit from special wards that are smaller, less crowded and have a higher rate of therapy contacts. We believe that psycho-education about psychopathology and trauma should be an early part of detoxification for this subgroup in addition to information on integrated treatment possibilities for addiction and comorbid disorders. Subsequent addiction treatment should integrate psychotherapy components for comorbid disorders such as posttraumatic stress disorder [46]. This might prevent, reduce or reverse ‘bounceback’ developments and increase the patients’ hope that a change of behavior is possible.

Our study has several limitations: first, it was underpowered and did not detect small effects. Secondly, the reported study was conducted with a selected sub-sample of the patients who received alcohol detoxification in our clinic. The participation rate of 45% was low. Thus, the sample was not representative. The most important reason for questionnaires not being returned was that some patients needed several days to regain sobriety and to overcome severe withdrawal effects so that they were about to leave the hospital before finalizing their study participation. In detoxification, this is inevitable due to short treatments. Short treatment stays are common in German detoxification and have been cited as problems by other studies [47]. However, we confirmed that patients who did not return the questionnaires did not differ from the ones who were included in the analysis. Third, our assessment did not include measures of posttraumatic symptom load, PTSD or other relevant psychopathology. We could not directly assess PTSD as establishing this diagnosis is not reliable during and in the weeks following after detoxification [28]. Our assessment did also not include a measure of baseline substance use severity; differences in this variable might explain that dropouts remained the same number of days in treatment than completers. In other studies, substance addicted patients with PTSD had a more severe substance use [15]; thus, the amount of baseline substance use might be a mediating variable between motivation to change and treatment outcome. Future studies should include a more detailed assessment of posttraumatic and other psychiatric symptoms as well as substance use severity. The study can also be criticized because of its restricted range of socio-demographic measures used to characterize individual study participants and of potential predictors of dropout such as level of education. In previous studies, a younger age predicted dropout [2]. In the current study, no such tendencies occurred and statistical methods confirmed that socio-demographic covariates seemed not to have a great influence on our outcome measure. However, a broader range of potential predictors of dropout needs to be assessed in future studies. Furthermore, the large number of statistical tests used in the current article increased the chance for a Type I error. Further limitations include the fact that the data had already been acquired four years ago and that the range of variables measuring treatment success was limited.

Future research should look into how patients with high and low Trauma Loads differ from each other. Not just different levels of psychopathology (as suggested above) but also different trajectories of addiction development and different types of substance use might exist. It is important to better understand exactly which behavioral changes the patients want to achieve or maintain: are they only related to substance use or do they encompass other goals such as relief of general psychological suffering? Future studies should also include a hypothesis on gender as moderating variable. A wide range of studies has shown that females have on average a higher risk of developing PTSD [48] and that Trauma Load is associated with more severe substance abuse among women [49]. Our data are compatible with the view that Trauma Load and female gender might interact and influence treatment completion; however, because we only had 15 female participants in our sample, this question could not be addressed here.

This study suggests that dropout from detoxification treatment more likely occurs in individuals with a high Trauma Load who have a low treatment motivation (especially Maintenance and Contemplation). While future empirical studies are certainly necessary to replicate and explain this finding, this study challenges the assumption that one kind of detoxification treatment fits all and warrants new thinking into individualized detoxification programs. It is urgently required that such interventions are further studied and that these become an integral part of detoxification. We believe that traumatized alcohol patients need special assistance during detoxification in order to prevent dropout and repeated admissions. In times of restricted public budgets such interventions will serve patients’ health and economic needs.

## Competing interests

The authors declare that they have no competing interests.

## Authors’ contributions

MO participated in the conception and design of the study, performed the statistical analysis and drafted the manuscript. PS participated in the design of the study, coordinated the data acquisition and helped to draft the manuscript. All authors read and approved the final manuscript.

## Supplementary Material

Additional file 1: Table S1 Multivariate model construction testing the moderation effect: The table shows five binary logistic regression models, dependent variable treatment completion. Click here for file

## References

[B1] Callaghan RC Cunningham JA Gender differences in detoxification: predictors of completion and re-admission J Subst Abuse Treat 2002 23 399 407 10.1016/S0740-5472(02)00302-1 12495802

[B2] Martinez-Raga J Marshall EJ Keaney F Ball D Strang J Unplanned versus planned discharges from in-patient alcohol detoxification: retrospective analysis of 470 first-episode admissions Alcohol Alcohol 2002 37 277 281 10.1093/alcalc/37.3.277 12003918

[B3] Braune NJ Schroder J Gruschka P Daecke K Pantel J Determinants of unplanned discharge from in-patient drug and alcohol detoxification: a retrospective analysis of 239 admissions Fortschr Neurol Psychiatr 2008 76 217 224 10.1055/s-2008-1038116 18415929

[B4] Reker T Richter D Batz B Luedtke U Koritsch HD Reymann G Short-term effects of acute inpatient treatment of alcoholics. A prospective, multicenter evaluation study Der Nervenarzt 2004 75 234 241 15021924 10.1007/s00115-003-1496-3

[B5] Prochaska JO DiClemente CC Stages and processes of self-change of smoking: toward an integrative model of change J Consult Clin Psychol 1983 51 390 395 6863699 10.1037//0022-006x.51.3.390

[B6] DiClemente CC Doyle SR Donovan D Predicting treatment seekers' readiness to change their drinking behavior in the COMBINE Study Alcohol Clin Exp Res 2009 33 879 892 10.1111/j.1530-0277.2009.00905.x 19320633 PMC2954369

[B7] Freyer-Adam J Coder B Ottersbach C Tonigan JS Rumpf HJ John U Hapke U The performance of two motivation measures and outcome after alcohol detoxification Alcohol and alcoholism 2009 44 77 83 19008551 10.1093/alcalc/agn088

[B8] Demmel R Beck B Richter D Reker T Readiness to change in a clinical sample of problem drinkers: relation to alcohol use, self-efficacy, and treatment outcome Eur Addict Res 2004 10 133 138 10.1159/000077702 15258444

[B9] Maisto SA Krenek M Chung T Martin CS Clark D Cornelius J A comparison of the concurrent and predictive validity of three measures of readiness to change alcohol use in a clinical sample of adolescents Psychol Assess 2011 23 983 994 21767028 10.1037/a0024136 PMC3433156

[B10] Field CA Adinoff B Harris TR Ball SA Carroll KM Construct, concurrent and predictive validity of the URICA: data from two multi-site clinical trials Drug Alcohol Depen 2009 101 115 123 10.1016/j.drugalcdep.2008.12.003 PMC3097110 19157723

[B11] Borsari B Murphy JG Carey KB Readiness to change in brief motivational interventions: a requisite condition for drinking reductions? Addict Behav 2009 34 232 235 10.1016/j.addbeh.2008.10.010 18990500 PMC2635060

[B12] Kulka RA Schlenger WE Fairbank JA Hough RL Jordan BK Marmar CR Weiss DS Trauma and the Vietnam War generation: report of findings from the National Vietnam Veterans Readjustment Study 1990 New York: Brunner/Mazel

[B13] Kessler RC Sonnega A Bromet E Hughes M Nelson CB Posttraumatic stress disorder in the National Comorbidity Survey Arch Gen Psychiatry 1995 52 1048 1060 10.1001/archpsyc.1995.03950240066012 7492257

[B14] Dom G De Wilde B Hulstijn W Sabbe B Traumatic experiences and posttraumatic stress disorders: differences between treatment-seeking early- and late-onset alcoholic patients Compr Psychiatry 2007 48 178 185 10.1016/j.comppsych.2006.08.004 17292709

[B15] Driessen M Schulte S Luedecke C Schaefer I Sutmann F Ohlmeier M Kemper U Koesters G Chodzinski C Schneider U Trauma and PTSD in patients with alcohol, drug, or dual dependence: a multi-center study Alcohol Clin Exp Res 2008 32 481 488 10.1111/j.1530-0277.2007.00591.x 18215214

[B16] Najavits LM Harned MS Gallop RJ Butler SF Barber JP Thase ME Crits-Christoph P Six-month treatment outcomes of cocaine-dependent patients with and without PTSD in a multisite national trial J Stud Alcohol Drugs 2007 68 353 361 17446974 10.15288/jsad.2007.68.353

[B17] Ouimette P Goodwin E Brown PJ Health and well being of substance use disorder patients with and without posttraumatic stress disorder Addict Behav 2006 31 1415 1423 10.1016/j.addbeh.2005.11.010 16380217

[B18] Chilcoat HD Breslau N Posttraumatic stress disorder and drug disorders: testing causal pathways Arch Gen Psychiatry 1998 55 913 917 10.1001/archpsyc.55.10.913 9783562

[B19] Del Gaizo AL Elhai JD Weaver TL Posttraumatic stress disorder, poor physical health and substance use behaviors in a national trauma-exposed sample Psychiatry research 2011 188 390 395 10.1016/j.psychres.2011.03.016 21481478

[B20] Jacobsen LK Southwick SM Kosten TR Substance use disorders in patients with posttraumatic stress disorder: a review of the literature Am J Psychiatry 2001 158 1184 1190 10.1176/appi.ajp.158.8.1184 11481147

[B21] Waldrop AE Back SE Verduin ML Brady KT Triggers for cocaine and alcohol use in the presence and absence of posttraumatic stress disorder Addict Behav 2007 32 634 639 10.1016/j.addbeh.2006.06.001 16863682

[B22] Ouimette P Moos RH Finney JW PTSD treatment and 5-year remission among patients with substance use and posttraumatic stress disorders J Consult Clin Psychol 2003 71 410 414 12699036 10.1037/0022-006x.71.2.410

[B23] Field CA Duncan J Washington K Adinoff B Association of baseline characteristics and motivation to change among patients seeking treatment for substance dependence Drug Alcohol Depen 2007 91 77 84 10.1016/j.drugalcdep.2007.05.009 17606335

[B24] Odenwald M Semrau P Reducing Dropout among Traumatized Alcohol Patients in Detoxification Treatment: A Pilot Intervention Study Eur Addict Res 2012 18 54 63 10.1159/000333336 22178762

[B25] Baron RM Kenny DA The moderator-mediator variable distinction in social psychological research: conceptual, strategic, and statistical considerations J Pers Soc Psychol 1986 51 1173 1182 3806354 10.1037//0022-3514.51.6.1173

[B26] Green BL Stamm BH Trauma History Questionnaire Measurement of Stress, Trauma and Adaptation 1996 Lutherville, MD: Sidran Press 366 369

[B27] Maercker A Deutsche Übersetzung des Trauma History Questionnaire Deutsche Übersetzung des Trauma History Questionnaire 200223615951

[B28] Liappas J Paparrigopoulos T Tzavellas E Christodoulou G Impact of alcohol detoxification on anxiety and depressive symptoms Drug Alcohol Depen 2002 68 215 220 10.1016/S0376-8716(02)00195-3 12234651

[B29] Neuner F Schauer M Karunakara U Klaschik C Robert C Elbert T Psychological trauma and evidence for enhanced vulnerability for posttraumatic stress disorder through previous trauma among West Nile refugees BMC Psychiatry 2004 4 34 10.1186/1471-244X-4-34 15504233 PMC529265

[B30] McConnaughy EA Prochaska JO Velcier WF Stages of Change in psychotherapy: measurement and sample profiles Psychotherapy 1983 20 368 375

[B31] Prochaska JO DiClemente CC Norcross JC In search of how people change. Applications to addictive behaviors Am Psychol 1992 47 1102 1114 1329589 10.1037//0003-066x.47.9.1102

[B32] Heidenreich T Hoyer J Fecht J Glöckner-Rist A, Rist F, Küfner H Veränderungsstadien-Skala (VSS) Elektronisches Handbuch zu Erhebungsinstrumenten im Suchtbereich (EHES), Version 100 2001 Mannheim: Zentrum für Umfragen, Methoden und Analysen

[B33] Fecht J Heidenreich T Hoyer J Lauterbach W Schneider R Veränderungsstadien bei stationärer Alkoholentwöhnung - Probleme der Diagnostik Verhaltenstherapie und Psychosoziale Praxis 1998 30 403 419

[B34] Carey KB Purnine DM Maisto SA Carey MP Assessing readiness to change substance abuse: A critical review of instruments Clin Psychol Sci Pract 1999 6 245 266 10.1093/clipsy.6.3.245

[B35] Project Match Research Group Matching Alcoholism Treatments to Client Heterogeneity: Project MATCH posttreatment drinking outcomes Journal of studies on alcohol 1997 58 7 29 8979210

[B36] Comrey AL Lee HB Elementary statistics: a problem-solving approach 1995 3 Dubuque, Iowa: Kendall/Hunt Pub. Co.

[B37] Jaccard J Interaction effects in logistic regression 2001 Thousand Oaks, Calif: Sage Publications

[B38] Langeland W Draijer N van den Brink W Psychiatric comorbidity in treatment-seeking alcoholics: the role of childhood trauma and perceived parental dysfunction Alcohol Clin Exp Res 2004 28 441 447 10.1097/01.ALC.0000117831.17383.72 15084902

[B39] Nelson TD Smith TR Pick R Epstein MH Thompson RW Tonniges TF Psychopathology as a Predictor of Medical Service Utilization for Youth in Residential Treatment J Behav Health Serv Res 2012 10.1007/s11414-012-9301-3 23229521

[B40] Odenwald M Steffen F Trauma load predicts first treatment in life among alcohol patients Can J Addict Med 2013 4 10

[B41] Curran GM Kirchner JE Worley M Rookey C Booth BM Depressive symptomatology and early attrition from intensive outpatient substance use treatment J Behav Health Serv Res 2002 29 138 143 10.1007/BF02287700 12032971

[B42] Stein BD Kogan JN Sorbero M Substance abuse detoxification and residential treatment among Medicaid-enrolled adults: rates and duration of subsequent treatment Drug Alcohol Depend 2009 104 100 106 10.1016/j.drugalcdep.2009.04.008 19481884 PMC2818065

[B43] Stein LA Minugh PA Longabaugh R Wirtz P Baird J Nirenberg TD Woolard RF Carty K Lee C Mello M Readiness to change as a mediator of the effect of a brief motivational intervention on posttreatment alcohol-related consequences of injured emergency department hazardous drinkers Psychol Addict Behav 2009 23 185 195 19586135 10.1037/a0015648 PMC2754149

[B44] Berman AH Forsberg L Durbeej N Kallmen H Hermansson U Single-session motivational interviewing for drug detoxification inpatients: effects on self-efficacy, stages of change and substance use Substance use & misuse 2010 45 384 402 10.3109/10826080903452488 20141454

[B45] Amaro H Chernoff M Brown V Revalo S Gatz M Does integrated trauma-informed substance abuse treatment increase treatment retention? J Community Psychol 2007 35 845 862 10.1002/jcop.20185

[B46] Schafer I Najavits LM Clinical challenges in the treatment of patients with posttraumatic stress disorder and substance abuse Curr Opin Psychiatry 2007 20 614 618 10.1097/YCO.0b013e3282f0ffd9 17921765

[B47] Schneider U Altmann A Baumann M Bernzen J Bertz B Bimber U Broese T Broocks A Burtscheidt W Cimander KF Comorbid anxiety and affective disorder in alcohol-dependent patients seeking treatment: the first Multicentre Study in Germany Alcohol and alcoholism 2001 36 219 223 10.1093/alcalc/36.3.219 11373258

[B48] Tolin DF Foa EB Sex differences in trauma and posttraumatic stress disorder: a quantitative review of 25 years of research Psychol Bull 2006 132 959 992 17073529 10.1037/0033-2909.132.6.959

[B49] Dom G DeWilde B Hulstijn W Sabbe B Traumatic experiences and posttraumatic stress disorders: differences between treatment-seeking early- and late-onset alcoholic patients Compr Psychiatry 2007 48 178 185 10.1016/j.comppsych.2006.08.004 17292709

