# Supplementary material for: Dropout among patients in qualified alcohol detoxification treatment: the effect of treatment motivation is moderated by Trauma Load
Source: Subst Abuse Treat Prev Policy. 2013 Mar 21;8:14. doi: 10.1186/1747-597X-8-14 (PMC3651403; doi:10.1186/1747-597X-8-14)
Supplement: Additional file 1: Table S1 — Multivariate model construction testing the moderation effect: The table shows five binary logistic regression models, dependent variable treatment completion. [file 1747-597X-8-14-S1.docx]

Additional file 1: Table S1: Multivariate model construction testing the moderation effect: The table shows five binary logistic regression models, dependent variable treatment completion. Models 1 to 4 are nested, model 5 is the final model. We report Odds Rations and 95% confidence intervals (in brackets) and Wald statistics and p values (in brackets).

|  | Model1 |  | Model2 |  | Model 3 |  | Model 4 |  | Final Model |  |
| --- | --- | --- | --- | --- | --- | --- | --- | --- | --- | --- |
|  | OR (CI95%) | Wald  (df, p) | OR (CI95%) | Wald  (df, p) | OR (CI95%) | Wald  (df, p) | OR (CI95%) | Wald  (df, p) | OR (CI95%) | Wald  (df, p) |
| Gender | 1.837  (0.528 – 6.385) | .915  (.339) | 1.990 (.515 – 7.689) | .996  (.318) | 2.909  (.638 – 13.267) | 1.902  (.168) | 5.064  (.763 – 33.597) | 2.823  (.093) | - | - |
| Age | .945  (.883 – 1.011) | 2.659  (.103) | .948  (.883 – 1.017) | 2.251  (.134) | .922  (.850 – 1.001) | 3.740  (.053) | .902  (.820 – .992) | 4.512  (.034) | - | - |
| Precontemplation | - | - | .963  (.721 – 1.287) | .065  (.798) | .942  (.698 – 1.272) | .151  (.697) | .850  (.509 – 1.419) | .387  (.534)- | - | - |
| Contemplation | - | - | 1.061  (.786 - 1.433) | .152  (.697) | 1.046  (.756 – 1.448) | .074  (.785) | 1.033  (.659 – 1.621) | .020  (.887) | - | - |
| Action | - | - | .922  (.738 – 1.152) | .513  (.474) | .999  (.780 – 1.279) | .000  (.8994) | 1.089  (.824 – 1.438) | .357  (.550) | - | - |
| Maintenance | - | - | 1.157  (.911 – 1.470) | 1.425  (.233) | 1.164  (.902 – 1.503) | 1.448  (.229) | 1.247  (.833 – 1.868) | 1.150  (.284) | - | - |
| THQ sum | - | - | - | - | .803  (.678 - .952) | 6.426  (.011) | .674  (.505 - .902) | 7.046  (.008) | .800  (.670 - .956) | 6.036  (.014) |
| Precontemplation x THQ-sum |  |  |  |  |  |  | .923  (.794 – 1.072) | 1.102  (.294) | - | - |
| Contemplation x THQ-sum |  |  |  |  |  |  | .924  (.808 – 1.057) | 1.332  (.248) | - | - |
| Action x THQ-sum |  |  |  |  |  |  | 1.124  (1.017 – 1.243) | 5.198  (.023) | - | - |
| Maintenance x THQ-sum | - | - | - | - | - | - | 1.120  (.970 – 1.294) | 2.372  (.124) | 1.050  (1.003 – 1.098) | 4.392  (.036) |
| Constant | .701 | 1.154  (.283) | .686 | 1.179  (.278) | .584 | 1.968  (.161) | .275 | 4.770  (.0239) | .767 | .756  (.385) |
| -2LL | 72.292^1^  (2; 0.183)^2^ |  | 67.858^1^  (6; 0.243) |  | 59.799^1^  (7; 0.025) |  | 48.183^1^  (11; .004) |  | 64.678^1^  (2; 0.004) |  |
| Nagelkere’s R^2^ | .080 |  | .180 |  | .337 |  | .513 |  | .245 |  |

^1^ comparison to model with constant only
